# Spatio-temporal evolution characteristics and influencing factors of environmental welfare performance in Chinese cities

**DOI:** 10.3389/fpubh.2025.1543745

**Published:** 2025-02-03

**Authors:** Yipeng Zhang, Meixia Wang

**Affiliations:** ^1^School of Ecnomics, Wuhan University of Technology, Wuhan, China; ^2^School of Economics and Management, Xi’an University of Technology, Xi’an, China

**Keywords:** environmental welfare performance, spatial distribution characteristics, spatial autocorrelation, hybrid-network-DEA model, regional difference

## Abstract

**Background:**

In the process of China’s urbanization, issues such as air pollution, water pollution, soil pollution, and noise pollution have become increasingly prominent, severely constraining the sustainable development of cities. The resultant decline in environmental welfare performance (EWP) not only affects residents’ quality of life but may also lead to public health issues, increasing healthcare costs, and subsequently impacting social stability and economic development.

**Methods:**

This paper incorporates factors closely related to environmental pollution, such as residents’ health and social welfare, into the analytical framework of environmental welfare performance. Using the Hybrid-Network-DEA model, we measure the EWP of 240 cities in China, and then investigate the spatial distribution characteristics and spatio-temporal evolution patterns of EWP. Finally, empirical testing of the factors influencing EWP is conducted using spatial econometric methods.

**Results:**

The overall level of EWP in 240 Chinese cities from 2004 to 2019 is relatively low, but it generally shows a wavy upward trend. Meanwhile, notable regional disparities exist in EWP, with the highest average performance in the east, followed by the west, and the lowest in the central. The main source of regional differences in EWP lies in inter-regional disparities. The greatest internal disparities are found in the east, while the largest inter-regional disparities are between the east and the west. A pronounced positive spatial autocorrelation is observed in the EWP among Chinese cities. Economic development, opening-up, financial development, digital infrastructure, and population density significantly promote the local EWP, whereas the industrial structure and transportation structure have exerted opposite effects. Additionally, the enhancement of EWP in neighboring regions is also notably facilitated by economic development, opening-up, financial development, and digital infrastructure. Within the three major regions, the direct and indirect effects of various influencing factors exhibit significant differences.

**Conclusion:**

Based on these insights, we suggest comprehensively improving environmental welfare efficiency, narrowing regional disparities, strengthening spatial agglomeration effects, optimizing industrial structure, and strengthening financial support and digital infrastructure construction.

## 1 Introduction

As the center of economic activities, urbanization forms economies of scale by concentrating population, industries, and resources, thereby enhancing production efficiency and economic benefits. Urbanization improves residents’ living conditions by offering more employment opportunities, higher-quality education and medical services, and a richer cultural life, thereby enhancing the quality of life (1). However, with the accelerated advancement of urbanization in China, the continuous expansion of urban scale and the high concentration of population have driven socio-economic development while also leading to a series of issues such as excessive resource consumption, intensified environmental pollution, and the shrinkage of ecological spaces (2, 3). These problems not only harm local public health and residents’ quality of life, but also severely constrain the improvement of urban environmental welfare performance (EWP) (4, 5). EWP is a multidimensional concept that encompasses not only traditional environmental quality indicators, such as air quality and water status, but also residents’ health levels, environmental satisfaction, and the contribution of the environment to the economy (6). This concept emphasizes that in the process of economic development, it is essential not only to pursue the speed and scale of economic growth but also to focus on environmental protection and the improvement of residents’ quality of life. Therefore, the core of EWP lies in achieving a balance and win-win situation among economic growth, environmental protection, and social welfare. Due to variations in regional economic development levels, resource endowments, and environmental regulatory policies in China (7), urban EWP may exhibit significant spatio-temporal heterogeneity. Scientifically assessing the spatio-temporal evolution characteristics of urban EWP and delving into its influencing factors are of great significance for formulating effective environmental policies and regional development strategies.

Current research on EWP primarily centers on the following two main areas: The First aspect concerts the connotation and evaluation methods of EWP: Early studies concentrated on assessing environmental quality, mostly using single pollutant indicators such as chemical oxygen demand (COD), PM_2.5_, PM_10_, SO_2_, NO_2_, and CO_2_ as proxy variables (8–12). However, the single-indicator measurement method has limitations, as it can only reflect one aspect of environmental performance and cannot comprehensively assess the overall level of environmental performance. Consequently, scholars have constructed comprehensive indicators to measure environmental performance, such as the air quality index, water quality index, ecosystem health index, and waste management indicators (13–17). Some scholars have also combined environmental performance with economic or other social performance indicators to construct the environmental performance index (EPI), which typically includes multiple dimensions, such as resource utilization efficiency, pollution control effectiveness, and ecosystem services (18). Meanwhile, with the popularization and application of input–output models, scholars commonly use DEA, SFA, and the Super-SBM model to measure environmental efficiency or environmental performance (19–22). Among these, DEA is often used to assess comprehensive efficiency, including factors such as energy consumption, waste generation, and pollution emissions. As research deepens, scholars have begun to integrate factors such as resident health and environmental satisfaction into the evaluation system of environmental performance, thus paying more attention to the multidimensionality of EWP. For instance, a Network DEA model was to proposed to evaluate regional development performance considering environmental pollution and health factors (23). An index system of environmental governance performance that includes three dimensions: environmental protection, environmental quality, and environmental welfare, is constructed and used the Generalized Entropy Index to decompose regional differences (24). The Hybrid-Network DEA model was adopted to measure EWP (25). This method not only considers traditional input–output factors but also incorporates resident health factors closely related to environmental pollution, making the measurement of EWP more aligned with the concept of harmonious coexistence between humans and nature.

The second aspect concerns the factors influencing EWP. Within the existing scholarly works, investigations into the determinants of EWP primarily concentrate on policy, economic, and social factors. Policy factors represent one of the crucial elements affecting EWP. By formulating and implementing environmental protection policies, governments can drive the in-depth development of environmental governance efforts, thereby enhancing EWP. For instance, pilot policies for low-carbon cities significantly boost EWP, and the impact of policy factors varies notably across regions (6). Environmental regulatory policies can improve EWP by fostering economic growth, promoting technological innovation, unleashing talent dividends, and optimizing industrial structures (25). The transparency of government environmental information disclosure is positively correlated with corporate environmental performance; disclosing more environmental information encourages companies to enhance their environmental performance (26). A carbon trading system can reduce carbon emissions per unit of output in the short term, maximizing residents’ welfare gains. Implementing a carbon tax policy is most effective in smoothing macroeconomic fluctuations, contributing to the stability of residents’ production and living. Economic factors including the economic development level, industrial structure, and resource utilization efficiency also exert significant influences on environmental governance performance. Regions with higher levels of economic development tend to exhibit better environmental governance performance, while areas with a higher proportion of heavy industries often face more severe environmental pollution problems, thereby affecting environmental governance performance (24). Local protectionism and market segmentation behaviors exacerbate resource misallocation, subsequently exerting negative impacts on EWP (27). Social factors are also among the key influences on EWP. For example, heightened environmental awareness and increased environmentally friendly behaviors among residents contribute to advancing environmental governance efforts, thereby enhancing EWP (25). At the micro level, for listed companies, stakeholder pressure significantly affects the environmental protection behaviors of manufacturing enterprises (28). Green capacity building, by enhancing employees’ environmental awareness and capabilities, has a notable impact on improving environmental performance (29). Supply chain volatility significantly inhibits corporate environmental performance; that is, greater supply chain volatility leads to lower environmental performance among manufacturing enterprises. Environmental collaboration with customers, green procurement, and sustainable innovation directly contribute to outstanding environmental performance (30).

Despite the achievements made in the measurement of EWP and its influencing factors in existing literature, there are still some deficiencies. Firstly, regarding the measurement of EWP, current studies mostly employ single-dimensional indicators or models, which fail to comprehensively reflect the essence of EWP. Secondly, existing research pays limited attention to the trends, regional disparities, and spatiotemporal evolution characteristics of EWP. Lastly, in terms of studying the influencing factors of EWP, current research tends to focus on the role of individual factors, lacking a thorough exploration of the combined effects of multiple factors. Therefore, this paper makes innovations in the following aspects:

(1) A multi-dimensional evaluation index system for EWP is constructed. Building on existing literature, this paper incorporates indicators such as economic development, environmental quality, resident health, and social welfare into the evaluation system of EWP, providing a more comprehensive reflection of the essence of EWP. Additionally, the hybrid network data envelopment analysis (Hybrid-Network-DEA) model is utilized to accurately measure the EWP of various cities.
(2) The spatiotemporal evolution characteristics of EWP are thoroughly explored. This paper not only focuses on the trends and regional disparities of EWP but also delves into its spatiotemporal evolution characteristics using exploratory spatial data analysis method (ESDAM). Through comparative analysis of EWP across different cities, the dynamic process of balancing environmental protection and economic development among cities is revealed.
(3) A comprehensive analysis of the influencing factors and mechanisms of EWP is conducted. When exploring the influencing factors of EWP, this paper not only considers the role of individual factors but also comprehensively analyzes the combined mechanisms of multiple factors. By employing spatial econometric models and conducting in-depth discussions on various influencing factors, the degree of impact of different factors on EWP is revealed. This provides valuable reference for governments to formulate scientific and reasonable environmental protection policies and promote green urban development.

## 2 Methods

### 2.1 Hybrid-network-DEA model

In the face of the dual challenges of economic development and environmental safeguarding, the research scope of environmental performance assessment should be expanded to encompass the idea of ecological civilization and the multidimensional pursuit of a better life by the public. However, most current evaluation indicators fail to adequately consider social welfare, which may lead to an overestimation of environmental performance. Amidst the ongoing construction of a Beautiful China and the modernization process of promoting harmonious coexistence between humans and nature, public attention to the green transformation of the economy and its impact on personal health and social well-being is increasing (6). Therefore, this paper incorporates factors closely related to environmental pollution, such as resident health and social welfare, into the analytical framework, and employs the Hybrid-Network-DEA model to construct an evaluation system of EWP that better aligns with the principle of harmonious coexistence between humans and nature.

**Figure 1:**
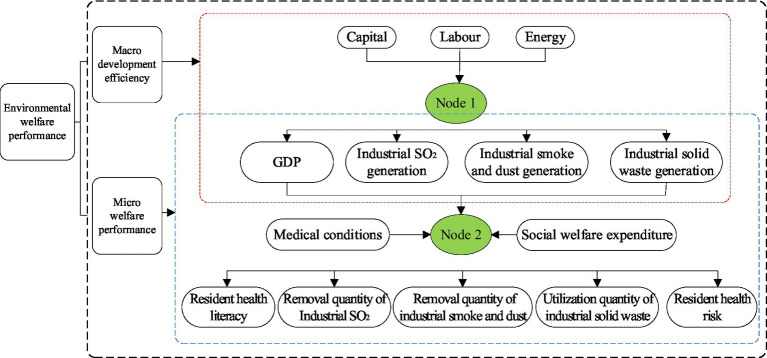
Hybrid-network-DEA model. The efficiency value of Node 1 is defined as macro-development performance, the efficiency value of Node 2 is defined as micro-welfare performance, and the combined efficiency value of the two represents EWP.

Given the limitations of traditional DEA models in revealing the internal details that influence overall efficiency changes within input–output systems, we design a Hybrid-Network-DEA model that integrates economic development, environmental pollution, and social welfare (25, 27). This model is a comprehensive evaluation approach that integrates traditional DEA methods with network structure analysis, offering multiple advantages. Firstly, it effectively addresses the internal structure of complex systems by decomposing decision-making units (DMUs) into multiple sub-processes or stages, enabling a more detailed assessment of the efficiency of each sub-process and its contribution to overall efficiency. The introduction of this network structure allows the model to uncover internal efficiency bottlenecks and optimization pathways, providing decision-makers with more targeted improvement recommendations. Secondly, the Hybrid-Network-DEA model can simultaneously consider multiple input and output indicators, making it suitable for multi-objective, multi-level efficiency evaluation problems, particularly in fields such as resource allocation and environmental performance assessment. In summary, the Hybrid-Network-DEA model, through its structured and comprehensive characteristics, provides powerful tool support for the efficiency evaluation and optimization of complex systems, as illustrated in Figure 1.

In Figure 1, the input factors for Node 1 are capital ( $Z_{0}^{1,1}$ ), labor ( $Z_{0}^{1,2}$ ), and energy ( $Z_{0}^{1,3}$ ), with the corresponding desired output being GDP ( $Z_{0}^{2,1}$ ) and the undesired outputs being industrial sulfur dioxide emissions ( $Z_{0}^{2,2}$ ), industrial smoke and dust emissions ( $Z_{0}^{2,3}$ ), and industrial solid waste generation ( $Z_{0}^{2,4}$ ). The efficiency value of Node 1 is defined as macro-development performance. Meanwhile, we consider the output factors of Node 1, along with medical conditions ( $Z_{0}^{2,5}$ ) and social welfare expenditures ( $Z_{0}^{2,6}$ ), as the input factors for Node 2. The corresponding desired outputs for Node 2 are residents’ health literacy ( $Z_{0}^{3,1}$ ), industrial sulfur dioxide removal ( $Z_{0}^{3,2}$ ), industrial smoke and dust removal ( $Z_{0}^{3,3}$ ), and industrial solid waste utilization ( $Z_{0}^{3,4}$ ), while the undesired output is residents’ health risk ( $Z_{0}^{3,5}$ ). Therefore, the efficiency value of Node 2 is defined as micro-welfare performance, and the comprehensive efficiency value of the two stages, Node 1 and Node 2, is defined as EWP.

When solving for the efficiency values of Node 1 and Node 2, given the close relationships between energy input and pollution, as well as between pollution and residents’ health risks, the simple assumption that inputs and outputs improve in equal proportions is not applicable here. Therefore, to accurately assess the efficiency of these two nodes, we need to combine the Hybrid Data Envelopment Analysis (Hybrid-DEA) model, which deals with undesired outputs, with the Network Data Envelopment Analysis (Network-DEA) model, thereby constructing the Hybrid-Network-DEA model. The specific expression is as follows:


(1) $$\left\{\begin{array}{l} \min\left(\theta_{1}+\theta_{2}\right)\\ \begin{array}{l} \sum_{j=1,\neq 0}^{n}\lambda_{j}^{1}z_{j}^{1,1}\leq \theta_{1}z_{0}^{1,1},\sum_{j=1,\neq 0}^{n}\lambda_{j}^{1}z_{j}^{1,2}\leq \theta_{1}z_{0}^{1,2},\\ \sum_{j=1,\neq 0}^{n}\lambda_{j}^{1}z_{j}^{1,3}\leq \theta_{1}z_{0}^{1,3}, \end{array}\\ \begin{array}{l} \sum_{j=1,\neq 0}^{n}\lambda_{j}^{1}z_{j}^{2,1}\geq \theta_{2}z_{0}^{2,1},\sum_{j=1,\neq 0}^{n}\lambda_{j}^{1}z_{j}^{2,2}\leq \theta_{2}z_{0}^{2,2},\\ \sum_{j=1,\neq 0}^{n}\lambda_{j}^{1}z_{j}^{2,3}\leq \theta_{2}z_{0}^{2,3},\sum_{j=1,\neq 0}^{n}\lambda_{j}^{1}z_{j}^{2,4}\leq \theta_{2}z_{0}^{2,4}, \end{array}\\ \lambda_{j}^{1}\geq 0,j=1,\cdots ,n,j\neq 0 \end{array}\right.$$

(2) $$\left\{\begin{array}{l} \begin{array}{l} \sum_{j=1,\neq 0}^{n}\lambda_{j}^{2}z_{j}^{2,1}\leq \theta_{2}z_{0}^{2,1},\sum_{j=1,\neq 0}^{n}\lambda_{j}^{2}z_{j}^{2,2}\leq \theta_{2}z_{0}^{2,2},\\ \sum_{j=1,\neq 0}^{n}\lambda_{j}^{2}z_{j}^{2,3}\leq \theta_{2}z_{0}^{2,3}, \end{array}\\ \begin{array}{l} \sum_{j=1,\neq 0}^{n}\lambda_{j}^{2}z_{j}^{2,4}\leq \theta_{2}z_{0}^{2,4},\sum_{j=1,\neq 0}^{n}\lambda_{j}^{2}z_{j}^{2,5}\leq \theta_{2}z_{0}^{2,5},\\ \sum_{j=1,\neq 0}^{n}\lambda_{j}^{2}z_{j}^{2,6}\leq \theta_{2}z_{0}^{2,6}, \end{array}\\ \begin{array}{l} \sum_{j=1,\neq 0}^{n}\lambda_{j}^{2}z_{j}^{3,1}\geq \theta_{2}z_{0}^{3,1},\sum_{j=1,\neq 0}^{n}\lambda_{j}^{2}z_{j}^{3,2}\geq \theta_{2}z_{0}^{3,2},\\ \sum_{j=1,\neq 0}^{n}\lambda_{j}^{2}z_{j}^{3,3}\geq \theta_{2}z_{0}^{3,3}, \end{array}\\ \begin{array}{l} \sum_{j=1,\neq 0}^{n}\lambda_{j}^{2}z_{j}^{3,4}\geq \theta_{2}z_{0}^{3,4},\\ \sum_{j=1,\neq 0}^{n}\lambda_{j}^{2}z_{j}^{3,5}\geq \theta_{2}z_{0}^{3,5}, \end{array}\\ \lambda_{j}^{2}\geq 0,j=1,\cdots ,n,j\neq 0 \end{array}\right.$$

(3) $$\begin{array}{l} \theta^{*}=\min\frac{1-\frac{1}{m}\left(\sum_{i=1}^{m_{1}}\left(S_{i}^{s-}/x_{i0}^{s}\right)+m_{2}\left(1-a\right)\right)}{1+\frac{1}{r}\left(\sum_{r=1}^{r_{1}}\left(S_{r}^{sg}/x_{r0}^{sg}\right)+r_{2}\left(1-a\right)\right)},\\ S_{i}^{s-}\geq 0,S_{r}^{sg}\geq 0,0\leq a\leq 1 \end{array}$$

Equations 1, 2 represent the constraint conditions for solving the efficiency values of Node 1 and Node 2, respectively, while Equation 3 calculates the efficiency value of a single node based on the Hybrid-Network-DEA model. Here, $m=m_{1}+m_{2}$ , *m*_1_ and *m*_2_ denote the number of divisible and indivisible input factors, respectively. Similarly, $r=r_{1}+r_{2}$ , *r*_1_ and *r*_2_ represent the number of divisible desired output factors and indivisible undesired output factors, respectively. $S_{i}^{s-}$ and $S_{r}^{sg}$ are the slack variables for divisible input factors and divisible desired output factors, respectively. $\lambda_{j}$ is the weight coefficient for the decision-making unit; $\theta_{1}$ is the calculated efficiency value for Node 1, and $\theta_{2}$ is the calculated efficiency value for Node 2.

### 2.2 Global Moran’s I

Global Moran’s I is an important indicator for measuring spatial correlation and aggregation. The specific calculation method is shown in Equation 4:


(4) $$I=\frac{n\overset{n}{\underset{i=1}{\sum }}\overset{n}{\underset{j=1}{\sum }}w_{ij}\left(x_{i}-\bar{x}\right)\left(x_{j}-\bar{x}\right)}{\overset{n}{\underset{i=1}{\sum }}\overset{n}{\underset{j=1}{\sum }}w_{ij}\overset{n}{\underset{i=1}{\sum }}{\left(x_{i}-\bar{x}\right)}^{2}}=\frac{\overset{n}{\underset{i=1}{\sum }}\overset{n}{\underset{j=1}{\sum }}w_{ij}\left(x_{i}-\bar{x}\right)\left(x_{j}-\bar{x}\right)}{S^{2}\overset{n}{\underset{i=1}{\sum }}\overset{n}{\underset{j=1}{\sum }}w_{ij}}$$

Where *n* is the sample size; *x_i_* and *x_j_* denote the observed value for spatial units *i* and *j*; *s* represents the standard deviation; and *W* is the spatial weight matrix.

Considering the missing data for some cities, if an adjacency weight matrix is constructed, it can not reflect the true spatial correlation. Therefore, we ultimately construct a geographic distance weight matrix and an economic-geographic distance matrix. The specific expressions for both are shown in Equations 5, 6:


(5) $$w_{ij}=\left\{\begin{array}{l} \frac{1}{d_{ij}},\;i\neq j\\ 0,\;i=j \end{array}\right.$$

(6) $$w_{ij}=\left\{\begin{array}{l} \frac{1}{\left|GDP_{i}-\right.\left.GDP_{j}\right|d_{ij}},\;i\neq j\\ 0,\;i=j \end{array}\right.$$

Where, *d_ij_* represents the distance between two regions; *GDP_i_* and *GDP_j_* represent the GDP of regions *i* and *j*.

### 2.3 Spatial econometric model

In this paper, the commonly used Spatial Durbin Model (SDM) is employed to explore the influencing factors of EWP, as presented in Equation 7.


(7) $$Y=\rho WY+\beta_{1}X+\beta_{2}WX+\varepsilon$$

Where *ρ* denotes the autocorrelation coefficient of the explained variable; *W* denotes the weight matrix. *Y* denotes the EWP; *X* denotes the influencing factors of EWP.

Furthermore, in SDM, since the right-hand side of the formula includes the spatially lagged terms of both the explanatory and explained variables, the coefficients obtained cannot directly reflect the magnitude of spatial spillover effects. Therefore, further decomposition is required to obtain the direct and indirect effects. By decomposition, Equation 7 can be transformed into the following two Equations 8, 9 (31):


(8) $$\left(I_{n}-\rho W\right)Y=\beta X+\theta WX+\varepsilon$$

(9) $$Y={\left(I_{n}-\rho W\right)}^{-1}+\left(I_{n}\beta +\theta W\right){\left(I_{n}-\rho W\right)}^{-1}X+{\left(I_{n}-\rho W\right)}^{-1}\varepsilon$$

Assuming $E\left(W\right)={\left(I_{n}-\rho W\right)}^{-1}$ , $V\left(W\right)=\left(I_{n}\beta +\theta W\right){\left(I_{n}-\rho W\right)}^{-1}$ , substitute them into Equation 9, combine it with Equation 10 to obtain Equation 11, and expand it to obtain Equation 12:


(10) $${\left(I_{n}-\rho W\right)}^{-1}=I_{n}+\rho W+\rho^{2}W^{2}+\rho^{3}W^{3}+\rho^{4}W^{4}+\ldots$$

(11) $$Y=\overset{n}{\underset{k=1}{\sum }}V_{k}\left(W\right)X_{k}+E\left(W\right)+E\left(W\right)\varepsilon$$

(12) $$\begin{array}{l} \left[\begin{array}{c} Y_{1}\\ Y_{2}\\ \vdots \\ Y_{n} \end{array}\right]=\left[\begin{array}{cccc} V_{k}{\left(W\right)}_{11} & V_{k}{\left(W\right)}_{12} & \ldots & V_{k}{\left(W\right)}_{1n}\\ V_{k}{\left(W\right)}_{21} & V_{k}{\left(W\right)}_{22} & \ldots & V_{k}{\left(W\right)}_{2n}\\ \vdots & \vdots & \ddots & \vdots \\ V_{k}{\left(W\right)}_{n1} & V_{k}{\left(W\right)}_{n2} & \ldots & V_{k}{\left(W\right)}_{nn} \end{array}\right]\left[\begin{array}{c} X_{1k}\\ X_{2k}\\ \vdots \\ X_{nk} \end{array}\right]\\ +E\left(W\right)+E\left(W\right)\varepsilon \end{array}$$

Where, $V_{k}\left(W\right)X_{ik}=\partial Y_{i}/\partial X_{ik}$ , $V_{k}\left(W\right)X_{jk}=\partial Y_{i}/\partial X_{jk}$ . $V_{k}\left(W\right)X_{ik}$ signifies the elements situated along the main diagonal of the coefficient matrix $X_{nk}$ , which indicate the direct effects. Additionally, it denotes the impact that the *k-*th explanatory variable in region *i* on the explained variable within the same region. $V_{k}\left(W\right)X_{jk}$ signifies the off-diagonal elements of the coefficient matrix $X_{nk}$ , which indicate the indirect effects. Additionally, it denotes the impact that the *k*-th explanatory variable in region *i* on the explained variable in a different region *j*. The sum of the indirect and direct effects gives the total effect.

### 2.4 Indicators and data sources

#### 2.4.1 Explained variables

According to Figure 1, the input indicators for Node 1 include capital, labor, and energy. Specifically, the capital stock is calculated using the fixed asset investment data of each city across the society (32). The labor is measured by the total number of employees in urban units, individuals, and private enterprises at the end of the year. Since energy consumption data are not currently published in urban yearbooks, we use total social electricity consumption as a proxy for energy input. The desired output indicator for Node 1 is urban GDP, while the undesired output indicators include industrial SO_2_ generation, industrial smoke and dust generation, and industrial solid waste generation.

We use the output indicators of Node 1, along with medical conditions and social welfare expenditures, as input indicators for Node 2. Among them, medical conditions are reflected by the number of hospital beds, and social welfare expenditures are represented by the sum of pension, social welfare relief, and social security expenditures. For the output variables corresponding to Node 2, considering the current lack of statistical data on life expectancy at the city level for each year in China, we adopt the per capita disposable income of urban residents as a proxy for residents’ health literacy, as it reflects their wealth status and higher income generally implies better health literacy (25). Meanwhile, the removal of industrial sulfur dioxide, industrial smoke and dust, and the utilization of industrial solid waste reflect the effectiveness of regional environmental governance, and are therefore included as desired outputs along with residents’ health literacy. Additionally, given that intensifying environmental pollution increases health risks for residents, making them more susceptible to illnesses and potentially leading to premature deaths, we select the annual number of deaths as an indicator to measure residents’ health risks and classify it as an undesired output.

#### 2.4.2 Independent variables

Drawing on the findings of some scholars and considering the characteristics of cities, we select seven key factors that may influence EWP, including economic development, industrial structure, opening-up, financial development, digital infrastructure, transportation structure, and population density. The specific explanations are as follows:

**Economic Development (RGDP)**: Economic development remains the prerequisite and guarantee for enhancing urban EWP. High-quality economic development contributes to achieving the sustainable development goal of “low consumption, high welfare.” As economic development trends upward, EWP also increases. This paper uses per capita GDP to measure urban economic development. Due to the lack of GDP deflators at the city level, the provincial GDP deflators are used to adjust the city’s GDP to constant 2004 prices (33).

**Industrial Structure (IS)**: The upgrading of industrial structure, such as the shift from high-pollution, high-consumption industries to services and technology-intensive industries, can reduce pollution emissions, improve the ecological environment, and thereby enhance ecological welfare performance. Conversely, an unreasonable industrial structure may lead to excessive resource consumption and increased environmental pollution, reducing ecological welfare performance. This paper measures the industrial structure using the proportion of the secondary industry in GDP (34).

**Opening-up (OPEN)**: On one hand, opening-up can introduce foreign capital and advanced technology, promote green technological progress, and improve pollution control capabilities, thereby exerting a positive effect on EWP. On the other hand, opening-up may also lead to the transfer of polluting industries to domestic markets, increasing environmental pollution and exerting a negative effect on EWP. Therefore, the ultimate impact of opening-up on EWP depends on the trade-off between positive and negative effects. This paper measures the degree of opening-up using the ratio of actual foreign investment to GDP (35).

**Financial Development (FD)**: On one hand, financial development can provide funding support for environmental projects and technological innovation, driving the growth of green industries, thereby reducing pollution emissions and promoting the improvement of environmental quality (36). On the other hand, if financial development excessively pursues short-term economic interests while ignoring environmental protection, it may lead to excessive capital flows into high-pollution, high-energy-consuming industries, exacerbating environmental problems and reducing EWP. This paper measures financial development using the ratio of financial institution deposits and loans to GDP (37).

**Digital Infrastructure (DINF)**: Digital infrastructure promotes the innovation and application of green technologies by enhancing data processing, network connectivity, and intelligence levels, reducing resource consumption and environmental pollution. At the same time, digital infrastructure can also improve management efficiency, optimize resource allocation, reduce unnecessary waste, and thereby improve environmental performance (38). Furthermore, digital infrastructure can drive the upgrading of the economic structure, promote sustainable development, and further enhance EWP. This paper measures digital infrastructure using the number of internet users and mobile phones per 100 people (39).

**Transportation Structure (TS)**: A diversified and efficient transportation structure can promote the development of environmentally friendly transportation modes, such as public transportation, railways, and water transportation, which significantly reduce carbon emissions and air pollution compared to private cars, helping to enhance environmental quality. Meanwhile, optimizing the transportation structure can also effectively alleviate urban traffic pressure, reduce traffic congestion and noise pollution, and enhance residents’ quality of life and happiness. This paper measures the transportation structure using road freight volume (40).

**Population Density (PD)**: On one hand, high population density may exacerbate resource consumption and environmental pollution, such as traffic congestion and increased pressure on waste disposal, thereby reducing EWP. On the other hand, high population density may also promote the effective utilization of public facilities and resource sharing, such as public transportation and green parks, which can help enhance environmental welfare. Therefore, population density’s impact on EWP depends on the effectiveness of urban planning, resource management, and environmental protection measures. This paper measures population density using the number of people per square kilometer (41).

#### 2.4.3 Data sources

Because of the absence of pertinent data for certain cities, this study has chosen 240 cities in China spanning the years from 2004 to 2019 as the subjects for measuring and analyzing their EWP. The data mainly come from the “China City Statistical Yearbook,” “China Energy Statistical Yearbook,” “China Environment Statistical Yearbook,” and the statistical yearbooks of various provinces and cities over the years. Although these data sources are authoritative and comprehensive, they still have certain limitations. Firstly, data in statistical yearbooks may suffer from inconsistencies in statistical standards or delays in updates, which can limit the comparability of data across different years or regions. Secondly, some indicators may have missing or incomplete data, particularly for certain small and medium-sized cities or underdeveloped regions, where records may not be comprehensive. This can affect the overall integrity and accuracy of the research. Therefore, this study used interpolation to supplement some of the missing data and excluded cities with severely insufficient data, focusing instead on 240 cities with relatively complete data.

## 3 Results and discussion

### 3.1 Measurement results of EWP

**Figure 2:**
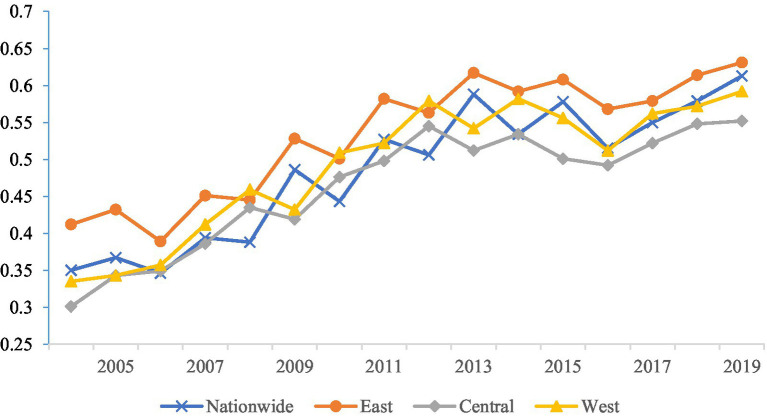
Trend of changes in urban EWP in China from 2004 to 2019.

As shown in Figure 2, we can clearly observe the significant trends in EWP across the country, as well as in the east, central, and west, from 2004 to 2019. Nationwide, EWP exhibited a wavy upward trend over these 16 years. Starting from 0.35 in 2004, it has grown continuously to reach 0.613 in 2019. The possible reasons lie in China’s gradual strengthening of the formulation and implementation of environmental protection policies during this period. For example, the construction of ecological civilization was set as an important goal in the “Eleventh Five-Year Plan” through the “Thirteenth Five-Year Plan,” which promoted the improvement of the environmental governance system and the implementation of the green development concept. Secondly, the transformation of the economic growth model played a crucial role, shifting from a high-pollution, high-energy-consumption extensive development to a green and low-carbon intensive development, which facilitated the improvement of resource utilization efficiency and the reduction of pollution emissions. Furthermore, the significant improvement in social welfare levels is also an important factor, including increased investments in education, healthcare, social security, and other fields, which directly enhanced residents’ quality of life and subsequently drove the improvement of EWP.

With respect to specific regions, our findings indicate that the east, being the most economically prosperous area in China, has consistently sustained a high level of EWP, exhibiting a gradual increase over time. From 0.412 in 2004 to 0.631 in 2019, it surpasses the average EWP of the central and west. Despite the growth rate in the east not being as pronounced as the national average, its stable development and high starting point make it a leader nationwide. This may be attributed to the east’s strong economic foundation, optimized industrial structure, which has allowed it to find a more balanced development path among environmental protection, social welfare, and economic development. Moreover, due to its economic prosperity and high education level, the public in the east has a relatively higher awareness of and participation in environmental protection.

Initially, the central’s EWP was relatively low, with an average of 0.464, significantly below the national urban average. However, over time, it has also shown a clear upward trend. Recently, the growth rate in the central has accelerated, gradually narrowing the gap with the east. This may be related to the central’s recent efforts in strengthening environmental protection, promoting industrial upgrading, and enhancing social welfare levels. The west’s initial EWP was similar to that of the central, with an average of 0.469, also significantly below the national urban average. However, as time passed, its growth rate gradually accelerated, particularly after 2010, when the performance value of the west significantly improved. This may be associated with the national government’s key support for the west, the region’s own resource endowments, and increased investments in environmental protection and social welfare recently.

### 3.2 Regional disparities and source decomposition of EWP

**Figure 3:**
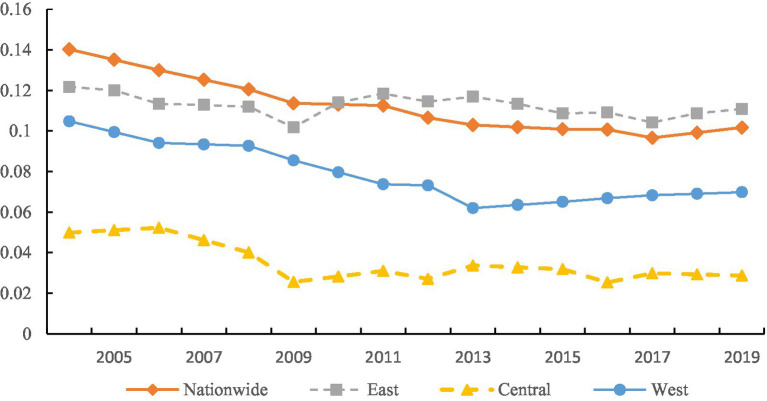
Overall and intra-regional disparities.

We explore the overall and regional disparities of China’s EWP, as illustrated in Figure 3. Specifically, the Gini coefficient declined from 0.14 in 2004 to 0.10 in 2009, representing a 27.5% decrease. The overall disparities showed a downward trend, indicating that the imbalanced development of China’s EWP has been effectively improved. In terms of intra-regional disparities, the Gini coefficients for the three major regions are, in descending order, the east, west, and central, with mean values of 0.102, 0.067, and 0.023, respectively. For the east, the Gini coefficient decreased from 0.111 in 2004 to 0.101 in 2009, then rose to 0.107 in 2013, followed by a fluctuating downward trend to 0.093 in 2017, and briefly rose again to 0.1 in 2019. Overall, it exhibited a trend of “steady decline - brief increase - fluctuating decline - brief increase.” The west, with 2013 as a turning point, showed a trend of first decreasing and then increasing. It steadily declined from 0.094 at the beginning of the sample period to 0.051 in 2013 and subsequently gradually rose to 0.059 in 2019. This may be attributed to the approval of the “12th Five-Year Plan for the Western Development” in 2012, which required accelerating the transformation of development modes and structural adjustments in the west, strengthening the national ecological barrier, and promoting resource conservation, intensive use, and recycling, thereby enhancing EWP. The central, with 2009 as a turning point, overall showed a development trend of “sharp decline - stable fluctuation.” It declined from 0.039 in 2004 to 0.015 in 2009 and subsequently fluctuated between 0.015 and 0.023.

In summary, the intra-regional disparities among the three major regions exhibit a pattern of east > west > central. A possible reason is the significant variation in EWP among cities in the east. Taking 2019 as an example, eastern cities such as Beijing, Shanghai, Tianjin, Guangzhou, Nanjing, and Hangzhou have EWP scores above 0.5, placing them in the “first tier.” Cities like Jilin, Shenyang, Yantai, Qingdao, Xi’an, and Baoji have scores above 0.4, forming the “second tier”; whereas Haikou, Yancheng, Qinhuangdao, Nanning, and Zhengzhou collectively constitute the “third tier,” which encompasses cities from the east, west, and central. This shows that all three tiers include eastern cities, which is the main reason for the large disparities in EWP within the east. The EWP in the central is generally comparable, with most cities having differences in EWP below 0.4, except for a few outliers, indicating smaller disparities among central cities.

**Figure 4:**
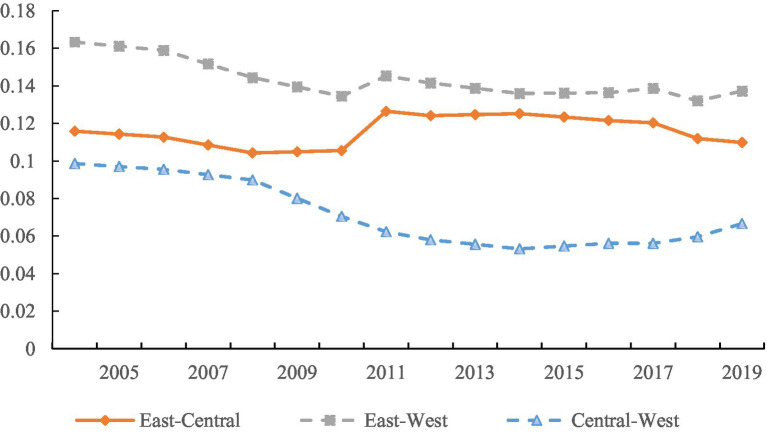
Inter-regional disparities.

As shown in Figure 4, the characteristics of inter-regional disparities in China’s EWP can be analyzed from two dimensions: the degree of disparity and the trend of disparity. Firstly, in terms of the degree of inter-regional disparity, the disparity between the east and the west is the largest, with an average Gini coefficient of 0.143; followed by the disparity between the east and the central, with an average Gini coefficient of 0.116; and the disparity between the central and the west is the smallest, with a Gini coefficient of 0.098. This indicates that during the sample period, the inter-regional disparities in China’s EWP were primarily driven by the disparities between two sets of regions: the east–west and east-central. Secondly, in terms of the trend of disparity, the Gini coefficients among all regions showed an overall declining trend during the sample period. Specifically, the Gini coefficient of central-west exhibited a steady decline, while the Gini coefficient of east–west showed a fluctuating downward trend. The Gini coefficient of east-central displayed a pattern of “slow decline - brief increase - fluctuating decline.” Among them, the Gini coefficient of central-west had the largest decrease, dropping from 0.143 in 2004 to 0.059 in 2019, a decrease of 58.7%; the decreases for the east–west and east-central pairs were smaller, at 15.96 and 5.21%, respectively. All the inter-regional Gini coefficients show varying degrees of decline, indicating that with China’s economic and social development, the regional disparities in EWP are overall narrowing.

The contribution rates of inter-regional disparity, intra-regional disparity, and hyper-variable density are shown in Table 1. From 2004 to 2019, the contribution rate of intra-regional disparity remained relatively stable, fluctuating between 22 and 25%. The contribution rate of inter-regional disparity gradually declined, from 64.811% in 2004 to 53.862% in 2019. The contribution rate of hyper-variable density showed an upward trend, increasing from 12.764% in 2004 to 21.519% in 2019. This indicates that inter-regional disparity plays a dominant role in overall disparity, followed by intra-regional disparity, while the contribution of hyper-variable density is relatively smaller. This trend clearly shows that the overall disparity in China’s EWP mainly stems from regional imbalances. From the perspective of contribution rate trends, both the contribution rate and absolute value of intra-regional disparity are relatively stable and may maintain this trend in the coming period. Although inter-regional disparity remains the main source of contribution, its importance is gradually diminishing; whereas the importance of hyper-variable density contribution is gradually increasing.

**Table 1:** Sources and contribution rates of regional disparities.

Year	Intra-regional disparity	Inter-regional disparity	Hyper-variable density	Contribution rate of intra-regional disparity (%)	Contribution rate of inter-regional disparity (%)	Contribution rate of hyper-variable density (%)
2004	0.031	0.091	0.018	22.425	64.811	12.764
2005	0.030	0.086	0.019	22.414	63.463	14.123
2006	0.029	0.081	0.020	22.404	62.115	15.481
2007	0.029	0.078	0.019	22.795	62.114	15.091
2008	0.028	0.075	0.018	23.185	62.114	14.701
2009	0.027	0.070	0.017	23.682	61.528	14.790
2010	0.026	0.068	0.018	23.543	60.238	16.219
2011	0.026	0.065	0.019	23.867	58.861	17.272
2012	0.026	0.061	0.020	24.192	57.485	18.324
2013	0.025	0.062	0.016	24.195	59.966	15.839
2014	0.024	0.061	0.016	24.035	60.278	15.687
2015	0.024	0.061	0.016	23.875	60.591	15.534
2016	0.024	0.059	0.018	24.000	58.574	17.427
2017	0.024	0.053	0.020	24.556	54.632	20.812
2018	0.024	0.054	0.021	24.588	54.247	21.165
2019	0.025	0.055	0.022	24.620	53.862	21.519

### 3.3 Spatial distribution characteristics of EWP

**Figure 5:**
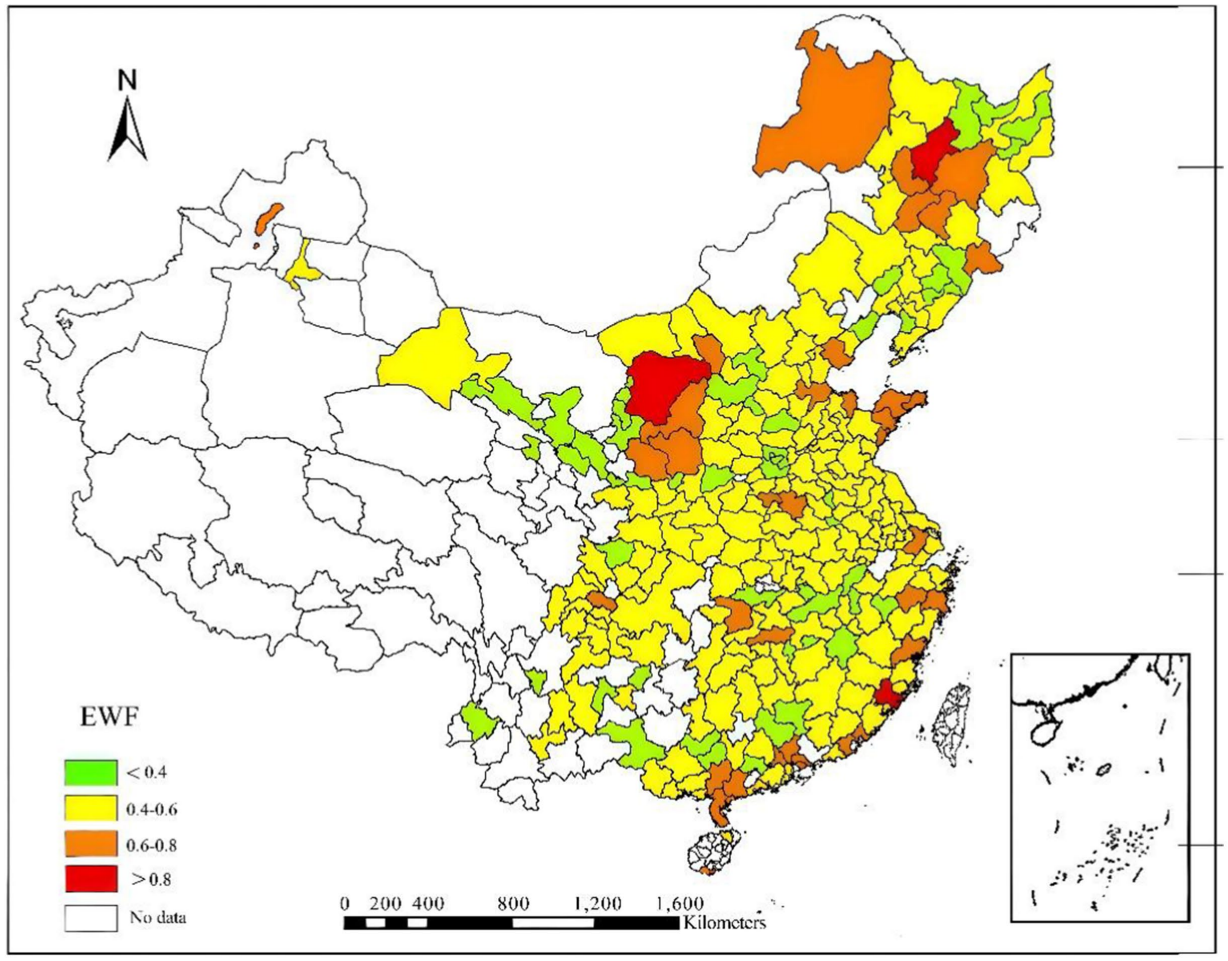
Overall distribution of urban EWP in China in 2019.

Based on the measured results, this paper depicts the overall spatial distribution of the average EWP across cities nationwide, as shown in Figure 5. The figure employs a color gradient to classify the EWP into four levels: from green to yellow, then to orange, and finally to red, representing a gradual increase in the value of urban EWP. Upon observation, it is found that most of the EWP values are densely distributed within the range of 0.4 to 0.6, while the areas with values below 0.4 and above 0.6 are relatively small. Upon further examination, it is evident that the EWP of cities located in the central and east primarily falls within the range of 0.4 to 0.5. Meanwhile, cities with higher EWP are scattered in individual areas of the east, central, and northeastern regions, as well as in some coastal provinces, specifically including Heilongjiang, Shandong, Shaanxi, Guangdong, Fujian, and Jiangsu. In terms of specific cities, Harbin, Suihua, Xiamen, Shenzhen, and Zhanjiang rank at the top in terms of EWP. Conversely, Hegang in Heilongjiang Province and Zhangye in Gansu Province are typical examples of cities with lower EWP. Overall, the EWP values of cities across the country are generally low, and there are significant differences in EWP values among various regions. Additionally, the spatial distribution of urban EWP exhibits a certain degree of agglomeration.

### 3.4 Results of spatial correlation test

Using Stata software and an economic geographical distance matrix, the global Moran’s I index for China’s urban EWP from 2004 to 2019 was calculated, as shown in Table 2. The Moran’s I values for EWP ranged from 0.096 to 0.204, all being greater than 0 and passing the significance test at the 1% level from 2004 to 2019. This suggests that there is a notable spatial clustering effect in the overall urban EWP of China. In other words, cities with higher EWP tend to be geographically close to each other, forming agglomerated areas with higher performance; correspondingly, cities with lower EWP also tend to be geographically adjacent, forming agglomerated areas with lower performance. The existence of this spatial agglomeration effect not only reflects the geographical proximity characteristics of China’s cities in terms of EWP but also provides useful clues for us to further explore and optimize urban EWP.

**Table 2:** Global Moran’s I index for EWP in China, 2004–2019.

Year	Moran’s *I*	Z	*p*-value
2004	0.107	2.922	0.003
2005	0.114	3.113	0.002
2006	0.116	3.156	0.002
2007	0.159	4.259	0.000
2008	0.130	3.500	0.000
2009	0.152	4.085	0.000
2010	0.160	4.298	0.000
2011	0.165	4.419	0.000
2012	0.149	3.980	0.000
2013	0.156	4.161	0.000
2014	0.096	2.605	0.009
2015	0.187	4.992	0.000
2016	0.164	4.384	0.000
2017	0.126	3.393	0.001
2018	0.155	4.142	0.000
2019	0.204	5.427	0.000

### 3.5 Regression results of full sample

#### 3.5.1 Model specification test

To ensure the reliability of the data analysis, we conduct a rigorous multicollinearity test on the selected variables using two key indicators: Variance Inflation Factor (VIF) and Tolerance (1/VIF). This is necessary because multicollinearity issues can distort model estimation results. As presented in Table 3, the VIF results for all variables are less than 5, and the Tolerance results are all greater than 0.1, confirming that there is no severe issue of multicollinearity among the variables.

**Table 3:** Multicollinearity test.

Variables	VIF	1/VIF
RGDP	1.54	0.648611
IS	1.18	0.846020
OPEN	1.32	0.757062
FD	1.01	0.994747
DINF	1.19	0.842886
TS	1.34	0.744613
PD	1.07	0.934401

Before conducting the regression, it is imperative to choose a suitable spatial econometric model by employing the LR test and Wald test to ascertain whether the Spatial Durbin Model (SDM) can be simplified to either the Spatial Lag Model (SLM) or the Spatial Error Model (SEM). As presented in Table 4, under the economic geographical distance matrix, both the LM test and LR test reject the null hypothesis at the 1% or 5% significance level, suggesting that the SDM is the most appropriate model. Additionally, since there are multiple estimation methods for panel data, to determine whether to use the random effects model or the fixed effects model, this paper conducts a Hausman test for verification. The results show that the Hausman test statistic is 115.21, with a *p*-value of 0.0000 < 0.01, indicating that the null hypothesis is rejected at the 1% significance level. Therefore, this study opts for the fixed effects model.

**Table 4:** LR test and Wald test.

Indicators	Statistics and *p*-values	Indicators	Statistics and *p*-values
LM Lag	1156.107*** (0.000)	LR spatial lag	31.126*** (0.002)
LM Lag (Robust)	25.260*** (0.000)	LR spatial error	26.264*** (0.000)
LM Error	1173.179*** (0.000)	Wald spatial lag	33.238*** (0.002)
LM Error (Robust)	34.289** (0.021)	Wald spatial error	31.362*** (0.000)
Hausman	115.21*** (0.000)		

#### 3.5.2 Analysis of regression results

In this paper, the SDM with both time and space fixed effects is selected for regression, and the results are compared with those of the Pooled OLS, SAR, and SEM. As shown in Table 5, the R^2^ value of SDM is 0.540, which is the highest among the four models, indicating that SDM has the best fitting results. Therefore, the regression results of the SDM are analyzed next. The Rho value is 0.336 and is significantly positive at the 1% level, indicating that EWP has evident spatial clustering features, aligning with the Moran’s I value computed previously. This means that the EWP of a city is significantly influenced by the EWP of nearby cities. Meanwhile, utilizing the methodology introduced by Lesage and Pace (2009), the impact effects are categorized into direct effect, indirect effect, and total effect, as shown in Table 6.

**Table 5:** Regression results of the SDM.

Variables	(1)	(2)	(3)	(4)
	OLS	SAR	SEM	SDM
RGDP	0.168*** (34.08)	0.134*** (27.05)	0.171*** (34.64)	0.145*** (19.49)
IS	−0.059*** (−5.15)	−0.063*** (−5.92)	−0.084*** (−7.05)	−0.073*** (−5.55)
OPEN	0.033** (2.09)	0.028* (1.88)	0.027* (1.88)	0.031** (2.12)
FD	0.061*** (12.98)	0.055*** (12.64)	0.075*** (14.14)	0.101*** (15.91)
DINF	0.005* (1.57)	0.010*** (3.3)	0.007** (2.11)	0.009*** (2.79)
TS	−0.002 (−0.44)	−0.002 (−0.56)	−0.002 (−0.07)	−0.052* (−1.69)
PD	0.011 (1.26)	0.009 (1.08)	0.011 (1.39)	0.010 (1.22)
RGDP×W				0.042*** (4.08)
IS×W				0.070*** (3.86)
OPEN×W				0.023 (0.80)
FD×W				0.077*** (9.99)
DINF×W				0.010* (1.89)
TS×W				−0.005 (−0.61)
PD×W				−0.004 (−0.29)
rho		0.297*** (17.21)		0.336*** (18.59)
lambda			0.345*** (18.89)	
R2	0.526	0.520	0.525	0.540
Log-likelihood		4744.033	4767.452	4824.205

***p < 0.01, **p < 0.05, *p < 0.1, with *t*-values in parentheses.

**Table 6:** Results of effect decomposition.

Variables	Direct effect	Indirect effect	Total effect
*RGDP*	0.118*** (13.91)	0.046*** (3.19)	0.164*** (12.84)
*IS*	−0.034*** (−2.81)	0.014 (0.57)	−0.020 (−0.82)
*OPEN*	0.033** (2.27)	0.126** (2.02)	0.159** (2.36)
*FD*	0.110*** (17.07)	0.066*** (7.90)	0.176*** (20.39)
*DINF*	0.011*** (3.21)	0.013* (1.69)	0.024** (2.42)
*TS*	−0.038*** (−3.46)	−0.000 (−0.03)	−0.038*** (−3.14)
*PD*	0.018** (2.37)	−0.005 (−0.22)	0.013 (1.16)

***p < 0.01, **p < 0.05, *p < 0.1, with *t*-values in parentheses.

The direct effect of industrial structure is −0.034 and is significant at the 1%, meaning that for every 1% increase in the proportion of the secondary industry, the local EWP will decrease by 0.034%. This is because the secondary industry typically includes a large number of heavy industries and energy-intensive industries, which often consume significant resources and emit pollutants during production, exerting considerable pressure on the environment. As the proportion of the secondary industry increases, resource consumption and pollution emissions may further intensify, leading to a decline in environmental quality and subsequently affecting EWP. The indirect effect of industrial structure is not significant.

The direct effect of opening-up is 0.033 and is significant at the 1%, indicating that for every 1% increase in the ratio of FDI to GDP, the local EWP will increase by 0.033%. This may be because opening-up has the capacity to draw in foreign investments and cutting-edge technology, promote green technological progress, and enhance pollution control capabilities, thereby exerting a positive effect on EWP. The indirect effect of opennes is 0.126 and is also significant at the 5% level, indicating that an increase in the local opening-up degree promotes the EWP of neighboring regions. Opening-up facilitates regional economic cooperation and exchanges, enabling neighboring regions to learn from and draw upon advanced environmental protection experiences and technologies, thereby enhancing their environmental management level.

The direct effect of financial development is 0.110 and is significant at the 1%, suggesting that it fosters the enhancement of local EWP. Financial development improves the efficiency of capital allocation, enabling more funds to flow into environmental protection industries and green technology fields, helping to reduce environmental pollution, improve resource utilization efficiency, and thereby enhance environmental quality. Simultaneously, financial advancement encourages the enhancement of environmental disclosure practices and regulatory frameworks, leading both enterprises and individuals to prioritize environmental conservation and sustainable development. These behavioral changes also contribute to enhancing EWP. The indirect effect of financial development is 0.066 and is significant at the 1%, meaning that financial development exerts a positive spatial spillover effect. As the scale of finance expands, financial institutions can provide more diversified financial products and services, including green credits and environmental protection investments, which help promote green development in neighboring regions. Meanwhile, financial development also facilitates inter-regional capital flows and information sharing, making it easier for neighboring regions to obtain financial and technical support, thereby improving environmental quality and enhancing EWP.

The direct effect of digital infrastructure is 0.011 and is significant at the 1%, indicating that for every 1% increase in the number of internet users and mobile phones per 100 people, the local EWP will increase by 0.011%. The improvement of digital infrastructure, such as the popularization of the internet and mobile phones, enhances the speed and quality of information flow, making the dissemination of environmental protection information and technologies more convenient, and promoting the enhancement of environmental awareness and changes in environmental behavior. Furthermore, the development of digital infrastructure also drives the innovation and upgrading of green industries, providing broader development spaces and more development opportunities for environmental protection industries. The indirect effect of digital infrastructure is 0.013 and passes the significance test at the 10%, indicating that the local digital infrastructure promotes the EWP of neighboring regions. The improvement of digital infrastructure enables information and knowledge to spread more rapidly and widely between regions, facilitating the learning and application of advanced environmental protection concepts and technologies by neighboring regions. At the same time, the digital infrastructure also strengthens inter-regional economic cooperation and exchanges, promoting the coordinated development of environmental protection industries.

The direct effect of transportation structure amounts to −0.038 and is statistically significant at the 1% level, suggesting that a 1% rise in road freight volume corresponds to a 0.038% decline in the local EWP. This is mainly because an increase in road freight volume is often accompanied by an intensification of issues such as tailpipe emissions and noise pollution, which directly negatively impact environmental quality and thereby reduce EWP. Additionally, an increase in road freight volume may result in issues like traffic congestion and damage to roads., further exacerbating environmental issues. The indirect effect of transportation structure is not significant, suggesting that the local transportation structure does not influence the EWP of neighboring regions.

The direct impact of population density stands at 0.018 and is notably significant at the 5% level, implying that a 1% surge in population density leads to a 0.018% increase in the local EWP. The reason may be that an increase in population density implies population agglomeration, which is often accompanied by improvements in resource utilization efficiency and the sharing of public facilities, thereby reducing resource waste and environmental pollution. At the same time, regions with higher population densities often have stronger environmental awareness and higher investments in environmental protection, driving the innovation of environmental technologies. The indirect effect of population density is not significant, suggesting that the local transportation structure does not influence the EWP of neighboring regions.

### 3.6 Robustness test

This study employs two methods for robustness testing. First, the original economic-geographical distance matrix is replaced with a geographical distance weight matrix, and the regression is performed again to obtain the effect decomposition results for the full sample and different regions, as shown in Table 7. We can see that except for a few individual variables, the direction and significance of the direct and indirect effects of various influencing factors are largely consistent with the previous regression results, indicating a considerable level of robustness. Second, partial samples with missing values are removed, and the SDM is used to perform the regression again, with the results presented in Table 7. It can be seen that by comparing the regression results before and after deleting the missing values, the coefficient signs, significance levels, and magnitudes of the independent variables remain largely consistent, demonstrating the robustness of the results. This also indicates that the method of handling missing values has a minimal impact on the research conclusions.

**Table 7:** Robustness test.

Variables	Geographical distance weight matrix			Deleting missing values		
	Direct effect	Indirect effect	Total effect	Direct effect	Indirect effect	Total effect
RGDP	0.117*** (12.76)	0.030 (0.25)	0.147*** (10.32)	0.109*** (9.72)	0.028 (0.24)	0.137*** (11.98)
IS	−0.049*** (−3.69)	0.042* (1.72)	−0.007 (−0.13)	−0.048*** (−3.28)	0.030 (1.12)	−0.018 (−0.42)
OPEN	0.031** (2.09)	0.120 (0.10)	0.151** (2.24)	0.031** (2.09)	0.125 (0.10)	0.156** (2.38)
FD	0.106*** (15.87)	0.081*** (13.42)	0.187*** (22.96)	0.105*** (14.76)	0.085*** (15.34)	0.190*** (21.48)
DINF	0.010*** (3.18)	0.020* (1.81)	0.030*** (2.76)	0.008** (2.14)	0.028* (1.86)	0.036** (2.41)
TS	−0.271** (−2.46)	−0.000 (−0.97)	−0.271** (−2.38)	−0.256** (−2.23)	−0.000 (0.99)	−0.256** (−2.16)
PD	0.100** (2.01)	−0.025 (−0.59)	0.075 (1.49)	0.117** (2.19)	−0.031 (0.64)	0.086* (1.73)

***p < 0.01, **p < 0.05, *p < 0.1, with *t*-values in parentheses.

### 3.7 Regression results for regional samples

The above section examines the factors influencing EWP from a national perspective. Taking into account that the influence of various factors on EWP may vary across different geographical locations, this paper categorizes 240 cities into three regions: east, central, and west. Similarly, using the economic-geographic distance matrix as the spatial matrix, a spatio-temporal dual fixed SDM is employed for regression, followed by effect decomposition, as presented in Table 8.

**Table 8:** Decomposition results of effects by region.

		RGDP	IS	OPEN	FD	DINF	TS	PD
East	Direct effect	0.196*** (9.46)	−0.197*** (−6.49)	0.109*** (3.49)	0.132*** (9.02)	0.021** (2.41)	0.010 (1.56)	0.031** (2.42)
	Indirect effect	−0.077*** (−2.57)	−0.164*** (−2.77)	0.335*** (2.68)	0.017 (0.89)	0.014** (2.32)	−0.004 (−0.22)	−0.005 (−0.17)
	Total effect	0.119*** (5.12)	−0.362*** (−6.14)	0.444*** (3.25)	0.149*** (6.62)	0.035** (2.16)	0.006 (0.31)	0.026 (0.82)
Central	Direct effect	0.248*** (12.48)	−0.146*** (−5.20)	−0.022 (−1.14)	0.025** (2.06)	0.017*** (2.67)	−0.021*** (−3.42)	−0.006 (−0.61)
	Indirect effect	−0.005 (−0.15)	0.166*** (2.84)	−0.038 (−0.53)	0.034*** (2.69)	0.043** (2.36)	0.005 (0.29)	−0.041 (−1.15)
	Total effect	0.243*** (8.64)	0.020 (0.37)	−0.060 (−0.75)	0.059*** (5.77)	0.060*** (3.18)	−0.016* (−1.79)	−0.047 (−1.16)
West	Direct effect	0.096*** (7.02)	0.030* (1.70)	0.024 (0.83)	−0.132*** (−9.34)	−0.011* (−1.68)	−0.052** (−2.51)	−0.065 (−1.10)
	Indirect effect	0.148*** (5.55)	−0.079* (−1.79)	0.057 (0.86)	0.092*** (5.00)	−0.027* (−1.90)	−0.004 (−0.52)	−0.391** (−2.41)
	Total effect	0.244*** (9.44)	−0.049 (−1.06)	0.081 (1.00)	−0.041** (−2.35)	−0.038** (−2.36)	−0.056** (−2.31)	−0.455*** (−2.57)

***p < 0.01, **p < 0.05, *p < 0.1, with *t*-values in parentheses.

Regarding economic development, the direct effect in the east is notably positive, whereas the indirect effect is markedly negative. This indicates that economic development in this region enhances the EWP of the city itself but hinders the EWP of adjacent cities. Within the central, the direct effect of economic development is distinctly positive, whereas the indirect effect lacks significance, suggesting that economic development in this area positively correlates with the EWP of the city itself but exerts no discernible influence on the EWP of neighboring cities. Within the west, both the direct and indirect impacts of economic development are notably positive, suggesting that the economic development not only boosts the EWP of the city itself but also favorably influences the EWP of nearby cities. Possible reasons are that in the east, due to its developed economy and intense resource competition, while local economic development can improve its own EWP, it may inhibit neighboring cities through resource siphoning, pollution transfer, etc. In the central region, economic development is in an upswing, with a primary focus on improving its own environmental conditions, thus having limited impact on surrounding cities. In the west, where the economy is relatively backward and there is ample room for development, economic growth can drive the joint improvement of EWP in both local and neighboring cities, achieving a win-win situation through regional cooperation, technology diffusion, and other means.

Regarding industrial structure, both the direct and indirect effects in the east are significantly negative, suggesting that an increase in the proportion of the secondary industry is not conducive to improving the EWP of the city itself and also inhibits the EWP of neighboring cities. Within the central, the direct effect is markedly negative, while the indirect effect is notably positive, suggesting that an increase in the proportion of the secondary industry inhibits the EWP of the city itself but stimulates the EWP of adjacent cities. Within the west, the direct effect is distinctly positive, whereas the indirect effect is markedly negative, suggesting that the industrial structure in this area enhances the EWP of the city itself but hampers the EWP of adjacent cities. Possible reasons are that the eastern region has high pollution emissions and significant resource consumption, which not only affect the EWP of the local city but also impact neighboring cities through pollution dispersion and other means. In the central region, industrial development in cities may have promoted industrial transfer and economic growth in neighboring cities, thereby enhancing their EWP. In the western region, cities may have inhibited the EWP of neighboring cities due to resource competition, pollution transfer, and other factors.

Regarding opening-up, both the direct and indirect effects in the east are notably positive, suggesting that FDI not only promotes the EWP of the city itself but also benefits the EWP of neighboring cities. In the central and west, neither the direct nor the indirect effects of opening-up are significant, indicating that FDI has no impact on the EWP of cities in these regions. Possible reasons lie in the strong ability of the eastern region to attract FDI. FDI has not only brought advanced technology and management experience, promoting environmental protection and sustainable development in the local city, but also enhanced the EWP of neighboring cities through technology spillovers, industrial linkages, and other effects. In contrast, the central and western regions are relatively backward, with lower levels of openness to the outside world and limited inflow of FDI, which may not have been effectively translated into improvements in EWP.

Regarding financial development, in both the east and central, both the direct and indirect impacts are notably positive, indicating that financial development in these two areas not only boosts the EWP of the city itself but also favorably affects the EWP of nearby cities. Within the west, the direct effect is significantly negative, while the indirect effect is positive, indicating that financial development in this region promotes the EWP of the city itself but inhibits the EWP of neighboring cities. Possible reasons are that due to the relatively mature financial systems in the eastern and central regions, the effective allocation of financial resources has not only promoted economic growth and environmental protection investments in the local cities, but also enhanced the EWP of neighboring cities through channels such as capital flows and technology dissemination. In contrast, the financial development in the western region is relatively lagging, and the concentration of financial resources may have intensified resource competition between the local city and its neighboring cities, resulting in improved EWP in the local city while suppressing that of neighboring cities due to resource scarcity.

Regarding digital infrastructure, both the direct and indirect influences in the east and central are distinctly positive, suggesting that in these two areas, the establishment of digital infrastructure not only enhances the EWP of the city itself but also positively impacts the EWP of adjacent cities. Conversely, in the west, both the direct and indirect effects are markedly negative, indicating that digital infrastructure in this area not only hinders the EWP of the city itself but also suppresses the EWP of neighboring cities. Possible reasons are that the relatively well-developed digital infrastructure in the eastern and central regions has facilitated information flow, technological innovation, and efficient resource allocation, not only enhancing the environmental management efficiency and the effectiveness of environmental investments in the local cities, but also driving the EWP of neighboring cities through technology spillovers and cooperative sharing mechanisms. In contrast, the digital infrastructure in the western region is relatively backward, and issues such as excessive resource concentration and environmental pollution may arise during its construction, which instead inhibit the EWP of both the local and neighboring cities.

With regards to transportation structure, neither the direct nor the indirect impacts in the east are significant, suggesting that an uptick in road freight volume in this area does not influence EWP. In the central and west, however, the direct effects are notably negative, while the indirect effects remain insignificant, indicating that a rise in road freight volume in these two regions suppresses the EWP of the city itself but does not affect the EWP of surrounding cities. Possible reasons are that in the eastern region, where the transportation network is well-developed, the environmental impact of increased road freight volume may be offset by other more efficient modes of transportation, thus having an insignificant effect on EWP. However, in the central and western regions, the increase in road freight volume is often accompanied by issues such as environmental pollution and traffic congestion, which inhibit EWP.

Regarding population density, the direct impact in the east is distinctly positive, whereas the indirect effect is not significant, suggesting that a rise in population density in this area enhances the EWP of the city itself but does not affect the EWP of adjacent cities. In the central, neither the direct nor the indirect effects are notable, indicating that an increase in population density in this region does not influence EWP. Conversely, in the west, the direct effect is insignificant, while the indirect effect is markedly negative, suggesting that while population density does not impact the EWP of the city itself, it hinders the EWP of neighboring cities. Possible reasons are that the dense population in the eastern region brings more economic activities and consumer demand, promoting the construction of urban environmental protection facilities and the application of environmental protection technologies, thereby enhancing EWP. In the central region, with a moderate population density, urban development and environmental pressure are relatively balanced, so changes in population density have no significant impact on EWP. In the western region, although the population density is low, population concentration may intensify resource competition and environmental pressure.

## 4 Conclusion and implications

### 4.1 Conclusion

As a multidimensional evaluation indicator, EWP not only focuses on environmental quality but also integrates social welfare and economic development, providing a comprehensive reflection of the balance between environmental protection, residents’ welfare, and economic sustainability in a region or country. Therefore, scientifically assessing the spatiotemporal evolution characteristics of urban EWP in China and delving into its influencing factors are of great significance for formulating effective environmental policies and regional development strategies. This paper incorporates factors closely related to environmental pollution, such as resident health and social welfare, into the analytical framework of EWP. The Hybrid-Network-DEA model is employed to measure the EWP of 240 cities in China. Additionally, the ESDAM is utilized to explore the spatial distribution characteristics and spatiotemporal evolution patterns of EWP. Finally, spatial econometric methods are adopted to empirically test the influencing factors of EWP. The primary conclusions reached are outlined below:

(1) From 2004 to 2019, the overall level of EWP across 240 cities in China was relatively low, but it exhibited an overall wavy upward trajectory. Meanwhile, significant regional variations in EWP were observed, with the east having the highest average, followed by the west, and the central having the lowest. However, in general, the EWP of the three regions is also steadily rising. Furthermore, the EWP of individual cities still needs further enhancement and improvement.
(2) The overall Gini coefficient of China’s EWP decreased from 0.14 in 2004 to 0.10 in 2009, showing a downward trend and indicating that regional disparities have been narrowing. Within the regions, the Gini coefficients for the three major regions are, in descending order, the east, west, and central, with mean values of 0.102, 0.067, and 0.023, respectively. In terms of inter-regional disparities, the greatest disparity is between the east and the west, followed by that between the east and the central, and the smallest disparity is between the central and west. Although inter-regional disparities remain the primary source of contribution, their significance is gradually diminishing; meanwhile, the importance of the contribution from hyper-variable density is gradually increasing.
(3) There is a significant positive spatial autocorrelation in the EWP of Chinese cities, indicating a spatial agglomeration effect. Cities with higher EWP are primarily clustered in economically prosperous regions like the eastern coast, including Xiamen, Shenzhen, Harbin, and Suihua. Cities with lower EWP are mainly concentrated in the west, such as Zhangye and Baoji.
(4) Across the full sample, the various influencing factors of urban EWP have different directions of effect. Among them, economic development, opening-up, financial development, digital infrastructure, and population density significantly promote the improvement of local EWP. However, industrial structure and transportation structure significantly inhibit local EWP. Economic development, opening-up, financial development, and the digital economy also exhibit positive spatial spillover effects, meaning these four factors significantly promote the improvement of EWP in neighboring areas. Industrial structure, transportation structure, and population density do not affect the EWP of neighboring areas.
(5) Within the three regions, the directions and effects of various influencing factors also show significant differences. Specifically: In all three regions, economic development promotes the improvement of local EWP. However, in the east, economic development inhibits the EWP of neighboring cities, while the opposite is true in the west. In the east and central, industrial structure inhibits the improvement of local EWP, while the opposite is true in the west. In the east, industrial structure inhibits the EWP of neighboring cities, while the central and west show the opposite trend. In the east, opening-up world not only promotes the EWP of the city itself but also benefits the EWP of neighboring cities. However, the impact of opening-up is not significant in the central and west. In the east and central, financial development promotes the improvement of local EWP, while the opposite is true in the west. Across all three regions, financial development promotes the improvement of EWP in neighboring cities. In the east and central, digital infrastructure construction not only promotes the improvement of local EWP but also benefits the EWP of neighboring cities, while the opposite is true in the west. In the central and west, the transportation structure inhibits the EWP of the city itself, while its impact is not significant in the east. In the east, population density promotes the improvement of local EWP, while in the west, it inhibits the EWP of neighboring cities.

### 4.2 Policy implications

(1) We should comprehensively enhance EWP and narrow regional disparities. In response to the overall low level of EWP and significant regional differences, a unified strategy for environmental protection and welfare improvement should be implemented nationwide, while emphasizing balanced development among regions. The east should continue to play a leading role, further enhancing EWP through technological and institutional innovations. The central and west need to increase investment in environmental protection, optimize resource allocation, and strive to enhance EWP to narrow the gap with the east and west. Therefore, it is necessary to further promote the in-depth implementation of policies such as the “Ecological and Environmental Zoning Management and Control Plan” and the “Action Plan for Advancing the Achievement of Carbon Peaking and Carbon Neutrality,” actively implement the requirements outlined in the “Opinions of the Central Committee of the Communist Party of China and the State Council on Comprehensively Promoting the Construction of a Beautiful China” and the “Healthy China 2030 Outline,” accelerate the modernization process of harmonious coexistence between man and nature, and make every effort to create a beautiful homeland with blue skies, green lands, and clear waters.
(2) Spatial agglomeration effects should be strengthened to promote coordinated regional development. Given the significant spatial autocorrelation of EWP, this characteristic should be fully utilized to facilitate coordinated development among regions. Eastern cities with higher EWP should strengthen cooperation with surrounding areas, assisting the central and west in improving their EWP through technology transfer and financial support. Meanwhile, the central and west should actively undertake industrial transfers from the east, but should be cautious about accepting high-pollution and high-energy-consuming industries. Additionally, cross-regional environmental protection cooperation mechanisms should be established to jointly address environmental pollution issues and achieve mutual improvement in EWP.
(3) Industrial structure should be optimized to promote green development. In response to the inhibitory effect of industrial structure on EWP, the optimization and upgrading of industrial structure should be accelerated to promote the development of green industries. Nationwide, support for green industries should be increased, encouraging enterprises to adopt clean production technologies, improve resource utilization efficiency, and reduce pollutant emissions. In the east, the expansion of high-pollution and high-energy-consuming industries should be strictly controlled, pushing industries toward high-end, intelligent, and green directions. In the central and west, guidance and support for industrial transformation and upgrading should be strengthened to avoid redundant construction and resource waste. Simultaneously, collaboration among industries ought to be strengthened in order to establish green industrial chains, thereby attaining a mutually beneficial scenario for both economic and environmental gains.
(4) Further investments in finance and digital infrastructure should be made to promote their balanced development nationwide. In the east, financial reforms ought to be further intensified, with the aim of optimizing the allocation of financial resources and enhancing finance’s capacity to cater to the real economy. Greater emphasis should be placed on the spillover effects of opening-up and financial development to promote high-quality regional economic development. In the central and west, financial infrastructure construction should be strengthened to improve the coverage and convenience of financial services. Simultaneously, the construction and upgrading of digital infrastructure should be enhanced to promote digital transformation and smart city construction, improving urban management and service levels. Additionally, differentiated policy measures should be adopted in response to the varying internal factors within each region.
(5) The eastern region, despite facing resource constraints, boasts a developed economy and strong governance capabilities. It should strengthen pollution control and ecological restoration, leveraging technological advantages to enhance resource utilization efficiency. The central region, with relatively abundant resources and a higher environmental carrying capacity, should optimize resource allocation. While undertaking industrial transfers from the east, it should prioritize green transformation, strengthen comprehensive watershed management and ecological barrier construction, and promote sustainable development in resource-based cities. The western region, rich in ecological resources but environmentally fragile, should focus on ecological protection, strictly control development intensity, develop eco-tourism and clean energy industries, improve ecological compensation mechanisms, and enhance environmental governance capabilities to achieve synergistic development of ecology and economy.

## Data Availability

The original contributions presented in the study are included in the article/supplementary material, further inquiries can be directed to the corresponding author.

## References

[1] Zhang ZH Zhao MC Zhang YP Feng YC (2023). How does urbanization affect public health? New evidence from 175 countries worldwide. Front Public Health 10:1096964. doi: 10.3389/fpubh.2022.1096964, PMID: 36684862 PMC9852986

[2] Gao YY Wang ST Zhang CX Xing CZ Tan W Wu HY (2023). Assessing the impact of urban form and urbanization process on tropospheric nitrogen dioxide pollution in the Yangtze River Delta. China Environ Pollut 336:122436. doi: 10.1016/j.envpol.2023.122436, PMID: 37640224

[3] Tian JQ Wang J Wang DL Fang CS (2024). Influence of urbanization on meteorological conditions and ozone pollution in the Central Plains urban agglomeration. China. Environ Pollut. 356:124290. doi: 10.1016/j.envpol.2024.124290, PMID: 38825221

[4] Zhang ZH Zhang GX Su B (2022). The spatial impacts of air pollution and socio-economic status on public health: empirical evidence from China. Socio Econ Plan Sci 83:101167. doi: 10.1016/j.seps.2021.101167

[5] Hua C Zhang ZH Miao JJ Han JW Zhu ZY (2025). Spatial coordination and industrial pollution of urban agglomerations: evidence from the Yellow River Basin in China. Expert Syst 42:e13548. doi: 10.1111/exsy.13548

[6] Ge LM Zheng HY Sun PB Zhu JL (2024). Low carbon with empowerment and efficiency: the impact of low-carbon city pilot policy on environmental welfare performance. Stat Res 41:100–14. doi: 10.19343/j.cnki.11-1302/c.2024.02.009

[7] Wang HP Zhang RJ (2022). Effects of environmental regulation on CO_2_ emissions: An empirical analysis of 282 cities in China. Sustain Prod Consump. 29:259–72. doi: 10.1016/j.spc.2021.10.016

[8] Xu Y Zhang WS Wang JH Ji SP Wang C Streets DG (2021). Investigating the spatially heterogeneous impacts of urbanization on city-level industrial SO_2_ emissions: evidence from night-time light data in China. Ecol Indic 133:108430. doi: 10.1016/j.ecolind.2021.108430

[9] Cheng SL Chen YT Wang KX Jia LJ (2024). Climate policy uncertainty influences carbon emissions in the semiconductor industry. Int J Prod Econ 278:109436. doi: 10.1016/j.ijpe.2024.109436

[10] Hua C Zhang ZH Miao JJ Sun HP Jia FL (2023). Do urban agglomeration planning policies promote the discharge reduction of industrial wastewater: evidence from the Yellow River Basin in China. Environ Res 239:117445. doi: 10.1016/j.envres.2023.117445, PMID: 37858686

[11] Liu H Gong YL Li SB (2024). Nonlinear impact and spatial spillover effect of new urbanization on PM_2.5_ from a multi-dimensional perspective. Ecol Indic 166:112360. doi: 10.1016/j.ecolind.2024.112360, PMID: 39822849

[12] Wu X Hao C Han C Ge CZ Duan XM Ren J (2025). Spatio-temporal coupling coordination analysis between local government environmental performance and corporate ESG performance. Environ Impact Asses Rev 110:107655. doi: 10.1016/j.eiar.2024.107655

[13] Schmidt S Laner D (2023). Environmental waste utilization score to monitor the performance of waste management systems: a novel indicator applied to case studies in Germany. Resour Cons Recy Adv 18:200160. doi: 10.1016/j.rcradv.2023.200160, PMID: 39822849

[14] Cai ZC Zhang Z Zhao F Guo XH Zhao JB Xu YY (2023). Assessment of eco-environmental quality changes and spatial heterogeneity in the Yellow River Delta based on the remote sensing ecological index and geo-detector model. Ecol Inform 77:102203. doi: 10.1016/j.ecoinf.2023.102203

[15] Dey S (2024). Urban air quality index forecasting using multivariate convolutional neural network based customized stacked long short-term memory model. Process Saf Environ 191:375–89. doi: 10.1016/j.psep.2024.08.076

[16] Wu HW Wu F Cai YM Li ZH (2024). Assessing the spatiotemporal impacts of land use change on ecological environmental quality using a regionalized territorial impact assessment framework. Sustain Cities Soc 112:105623. doi: 10.1016/j.scs.2024.105623

[17] Wu YD Peng CR Li GB He F Huang LC Sun XQ (2024). Integrated evaluation of the impact of water diversion on water quality index and phytoplankton assemblages of eutrophic Lake: a case study of Yilong Lake. J Environ Manag 357:120707. doi: 10.1016/j.jenvman.2024.120707, PMID: 38554455

[18] Latif N (2022). Comprehensive environmental performance index (CEPI): An intuitive indicator to evaluate the environmental quality over time. Environ Res Commun 4:075016. doi: 10.1088/2515-7620/ac8338

[19] Fazio G Maioli S Rujimora N (2024). Building back greener, levelling-up or both? An assessment of the economic and environmental efficiency transition of UK regions. Pap Reg Sci 103:100053. doi: 10.1016/j.pirs.2024.100053

[20] Li KJ Zou Z Zhang Y Shuai CY (2024). Assessing the spatial-temporal environmental efficiency of global construction sector. Sci Total Environ 951:175604. doi: 10.1016/j.scitotenv.2024.175604, PMID: 39173753

[21] Li ZY Kang S (2024). Towards sustainable development goals: An analysis of environmental efficiency and the impacts of self-purification capacity across diverse income levels. Environ Res 261:119678. doi: 10.1016/j.envres.2024.119678, PMID: 39067804

[22] Lin XY Jing XD Cheng F Wang M (2024). Exploration of port environmental efficiency measurement and influential factors in the Yangtze River Delta pilot free trade zone. Mar Pollut Bull 206:116766. doi: 10.1016/j.marpolbul.2024.116766, PMID: 39094282

[23] Kao C (2009). Efficiency decomposition in network data envelopment analysis: a relational model. Eur J Oper Res 192:949–62. doi: 10.1016/j.ejor.2007.10.008

[24] Guan YB (2021). Study on regional differences of environmental governance performance in the Yangtze River Economic Belt:decomposition based on generalized entropy index. J Tech Econ Manag 40:547–54.

[25] Shao S Ge LM Zhu JL (2024). How to achieve the harmony between humanity and nature: environmental regulation and environmental welfare performance from the perspective of geographical factors. J Manag World 8:88–102. doi: 10.19744/j.cnki.11-1235/f.2024.0088

[26] Tan X Liu H (2024). Corporate environmental performance under the pressure of government environmental information disclosure. Financ Res Lett 69:106152. doi: 10.1016/j.frl.2024.106152

[27] Song ML Jin PZ (2016). Regional protection, resource misallocation and environmental welfare performance. Econ Res J 51:47–61.

[28] Shahzad MF Xu S An X Asif M Jafri MAH (2024). Effect of stakeholder pressure on environmental performance: do virtual CSR, green credit, environmental and social reputation matter? J Environ Manag 368:122223. doi: 10.1016/j.jenvman.2024.122223, PMID: 39163671

[29] Tahir AH Umer M Nauman S Abbass K Song H (2024). Sustainable development goals and green human resource management: a comprehensive review of environmental performance. J Environ Manag 370:122495. doi: 10.1016/j.jenvman.2024.122495, PMID: 39332294

[30] Sadiq M Nawaz MA Sharif A Hanif S (2024). Bridging green supply chain practices and environmental performance in Chinese semiconductor sector: with the role of energy efficiency and green HRM. Int J Prod Econ 277:109381. doi: 10.1016/j.ijpe.2024.109381

[31] Lesage JP Pace RK (2009). A sampling approach to estimate the log determinant used in spatial likelihood problems. J Geogr Syst 11:209–25. doi: 10.1007/s10109-009-0087-7, PMID: 39823079

[32] Zhang J Wu GY Zhang JP (2004). The estimation of China’s provincial capital stock: 1952-2000. Econ Res J 10:35–44.

[33] Dong YJ Wang FY (2024). Wang KY the effect and its mechanism of turning county (county-level city) into urban district on the economic development in regions with lagging urbanization: evidence from northeast and Southwest China. Habitat Int 151:103141. doi: 10.1016/j.habitatint.2024.103141

[34] Zhang QM Zhao X (2024). Can the digital economy facilitate the optimization of industrial structure in resource-based cities? Struct Change Econ D 71:405–16. doi: 10.1016/j.strueco.2024.08.010

[35] Wang L Ramsey TS (2024). Digital divide and environmental pressure: a countermeasure on the embodied carbon emissions in FDI. Technol Forecast Soc Change 204:123398. doi: 10.1016/j.techfore.2024.123398

[36] Zhang ZH Zhang YP Zhao MC Muttarak R Feng YC (2023). What is the global causality among renewable energy consumption, financial development, and public health? New perspective of mineral energy substitution. Resour Policy 85:104036. doi: 10.1016/j.resourpol.2023.104036

[37] Li GC Wei WX (2021). Financial development, openness, innovation, carbon emissions, and economic growth in China. Energy Econ 97:105194. doi: 10.1016/j.eneco.2021.105194

[38] Zhang ZH Zhang YP Wu HB Song SF Pan YX Feng YC (2024). Dual effects of automation on economy and environment: evidence from A-share listed enterprises in China. China Econ Rev 88:102308. doi: 10.1016/j.chieco.2024.102308

[39] Tang KJ Yang GY (2023). Does digital infrastructure cut carbon emissions in Chinese cities? Sustain Prod Consump 35:431–43. doi: 10.1016/j.spc.2022.11.022

[40] Liu HY Zhang ZQ Cai XZ Wang DW (2024). Liu M investigating the driving factors of carbon emissions in China's transportation industry from a structural adjustment perspective. Atmos Pollut Res 15:102224. doi: 10.1016/j.apr.2024.102224

[41] Liang DZ Lu HW Guan YL Feng LY He L Qiu LH (2023). Population density regulation may mitigate the imbalance between anthropogenic carbon emissions and vegetation carbon sequestration. Sustain Cities Soc 92:104502. doi: 10.1016/j.scs.2023.104502

